# DNA-Encoded Flagellin Activates Toll-Like Receptor 5 (TLR5), Nod-like Receptor Family CARD Domain-Containing Protein 4 (NRLC4), and Acts as an Epidermal, Systemic, and Mucosal-Adjuvant

**DOI:** 10.3390/vaccines1040415

**Published:** 2013-09-25

**Authors:** Sanna Nyström, Andreas Bråve, Tina Falkeborn, Claudia Devito, Björn Rissiek, Daniel X. Johansson, Ulf Schröder, Satoshi Uematsu, Shizuo Akira, Jorma Hinkula, Steven E. Applequist

**Affiliations:** 1 Center for Infectious Medicine, F59, Department of Medicine, Karolinska Institutet, Karolinska University Hospital Huddinge, Stockholm 141 86, Sweden; 2 Department of Preparedness, Swedish Institute for Infectious Disease Control, Stockholm 171 82, Sweden; 3 Division of Molecular Virology, Department of Clinical and Experimental Medicine, Linköping University, Linköping 581 85, Sweden; 4 Institute of Immunology, University Medical Center Hamburg-Eppendorf, Hamburg D-20246, Germany; 5 Department of Microbiology, Tumor and Cell Biology, Karolinska Institutet, Nobels väg 16, Stockholm 171 77, Sweden; 6 Latour AB, Stockholm 171 21, Sweden; 7 Division of Innate immune regulation, International Research and Development Center for Mucosal Vaccine, Institute for Medical Science, The University of Tokyo, Tokyo 108-8639, Japan; 8 Department of Host of Defense, Research Institutet for Microbial Diseases, Osaka University, Osaka 565-0871, Japan

**Keywords:** adaptive immunity, DNA adjuvant, flagellin, NLRC4, TLR5

## Abstract

Eliciting effective immune responses using non-living/replicating DNA vaccines is a significant challenge. We have previously shown that ballistic dermal plasmid DNA-encoded flagellin (FliC) promotes humoral as well as cellular immunity to co-delivered antigens. Here, we observe that a plasmid encoding secreted FliC (pFliC(-gly)) produces flagellin capable of activating two innate immune receptors known to detect flagellin; Toll-like Receptor 5 (TLR5) and Nod-like Receptor family CARD domain-containing protein 4 (NRLC4). To test the ability of pFliC(-gly) to act as an adjuvant we immunized mice with plasmid encoding secreted FliC (pFliC(-gly)) and plasmid encoding a model antigen (ovalbumin) by three different immunization routes representative of dermal, systemic, and mucosal tissues. By all three routes we observed increases in antigen-specific antibodies in serum as well as MHC Class I-dependent cellular immune responses when pFliC(-gly) adjuvant was added. Additionally, we were able to induce mucosal antibody responses and Class II-dependent cellular immune responses after mucosal vaccination with pFliC(-gly). Humoral immune responses elicited by heterologus prime-boost immunization with a plasmid encoding HIV-1 from gp160 followed by protein boosting could be enhanced by use of pFliC(-gly). We also observed enhancement of cross-clade reactive IgA as well as a broadening of B cell epitope reactivity. These observations indicate that plasmid-encoded secreted flagellin can activate multiple innate immune responses and function as an adjuvant to non-living/replicating DNA immunizations. Moreover, the capacity to elicit mucosal immune responses, in addition to dermal and systemic properties, demonstrates the potential of flagellin to be used with vaccines designed to be delivered by various routes.

## 1. Introduction

DNA-vaccines are promising tools with great potential for combating infectious disease. Non-living/replicating DNA vaccines have several advantages over living viral delivery vectors, such as lower production costs, increased stability, a higher overall safety profile, and recent evidence indicates that they can provide humans with protective immunity to viral infection [1]. However, living viral vectors used in DNA vaccine settings (such as Adenovirus) can still elicit stronger immune responses in humans than naked DNA. Yet in the case of adenovirus, evidence suggests that they may not promote the desired immune responses to the recombinant antigen. As results from clinical trials show, the use of a viral vector can, possibly as a consequence of the anti-vector immunity, potentially even enhance the risk of infection with certain pathogens [2]. These observations emphasize the critical need to continue research on methods for adjuvanting minimal, non-living/replicating DNA vaccines.

There are many approaches to improving the efficacy of plasmid DNA vaccines such as choice of delivery method, modifications of antigen location/stability/presentation, and the use of immunopotentiators [3]. Here, we investigate a formulation-compatible immunopotentiating adjuvant, with the potential to activate innate and adaptive immune responses through Toll-like Receptor 5 (TLR5) and/or possibly Nod-like Receptor (NLR) family members NLRC4 and Naip5 [4]. This approach employs plasmid DNA encoding a secreted form of flagellin (FliC) from *Salmonella typhimurium* as an adjuvant in DNA vaccinations. This adjuvant allows mammalian cells to create an environment of sterile-inflammation, thus mimicking natural infection in a safe manner and promoting adaptive immune responses to co-delivered DNA-encoded antigens [5]. This approach is unique in that it uses a plasmid-encoded agonist of innate immune receptors to activate a large variety of molecules capable of promoting adaptive immunity, unlike many other approaches which use single cytokines or chemokines [3]. A major benefit of this system is that it works without physically linking the antigen to flagellin. This ensures that the antigen is properly folded and processed and constitutes a major practical advantage as the system is flexible and can be applied with ease to various antigens without the need for time-consuming development of fusion-constructs. Recombinant flagellin produced in bacteria is currently being used by many as an experimental adjuvant to promote humoral and cellular immunity against microbial pathogens [6,7,8]. However, the use of flagellin in protein-form presents formulation and stability issues with non-living/replicating DNA vaccines such as plasmids.

In previous work, we vaccinated mice epidermally, using a gene-gun, with a transmembrane-anchored form of flagellin (pFliC-Tm) and secreted ovalbumin (pOVA). We observed significant increases in antigen-specific serum IgG levels compared to pOVA alone as well as strong antigen-specific CD4^+^/8^+^ cellular immune responses [5]. Importantly, we also showed that the pFliC-Tm adjuvant delivered with a DNA-encoded nucleoprotein gene from Influenza A resulted in a strong antigen-specific CD4^+^/8^+^ cellular immune response which correlated with protection from lethal virus infection [5]. This work demonstrated that pFliC-Tm acts as an adjuvant when delivered dermally however it is not clear whether this is the optimal route for eliciting the broadest or strongest immune responses. Additionally, not all DNA vaccination approaches are applied dermally therefore further studies of adjuvant effects induced by various delivery routes are warranted.

The HIV-1 pandemic has been estimated to have according with WHO/UNAIDS reports been spread globally and infected individuals exist in all countries in the world. So far, only a few experimental vaccine studies have shown promising and protective results in clinical trials. Thus there is a continued need to find more efficient vaccination strategies to provide protective immunity against the infection. Since, the main route of infection is via sexual transmission and via mucosal transmission such as breast-feeding, a vaccine that can provide mucosal immunity would be desirable. However, mucosal vaccines against infectious disease are few, and only polio, influenza, rotavirus, *S. typhi*, and *V. cholerae* have commercially available vaccines [9]. There are numerous adjuvants now found to promote mucosal immune responses, some of them lipid based, however none of these are in themselves plasmid-DNA based technologies [10].

Here, we studied if a plasmid vector expressing secreted FliC (pFliC(-gly)) activates TLR5- and NLRC4/Naip5-specific innate immune responses and acts as an adjuvant to plasmid-encoded antigen by three different routes representative of dermal, systemic, and mucosal locations. Additionally, we performed intranasal mucosal immunizations using plasmid encoding the clinically relevant HIV-1 antigen gp160 followed by recombinant HIV-1 protein booster. The ability of pFliC adjuvant to enhance HIV-1 gp160 envelope immune responses at mucosal and systemic compartments was also investigated.

## 2. Experimental

### 2.1. Cloning of Vaccination Expression Constructs and Molecular Mechanism Constructs

pOVA and pFliC-Tm(-gly) have been described previously [5]. pFliC-Tm(-gly) was subjected to site-directed mutagenesis to insert two translational stop-codons after AA 459 of FliC(-gly) (AA numbering is based on GenBank Accession #D13689). Changes of all constructs were confirmed by DNA sequencing. Variants of FliC(-gly) were created for testing the molecular mechanism of FliC(-gly) detection *in vitro*. First, to make a cytoplasmically-expressed FliC(-gly) the secretion leader sequence of FliC(-gly) was removed from the pFliC(-gly) vector by PCR amplification using the primers 5'-ccaggttccAATCTTATGTatccatatgatgttccagattatgct-3' and 5'-GCAGCCGCGGATCCCGGGGTACCTATCGCAGTAAAGAGAGGACGTTTTGCGG-3' with the pFliC(-gly) template encoding a starting methionine, HA-tag, and complete FliC(-gly) open-reading frame (ORF) without the secretion leader sequence. Full-length products were digested with *Hin*d III/*Bam*H I and ligated into pcDNA 3.1/Zeo(+) prepared with HindIII/BamHI to make pcFliC(-gly). To remove the COOH-terminal 34 amino-acids of FliC a section of the FliC(-gly) gene encoding AA282 to 461 residing on a *Bsr*G I/*Xho* I fragment (5'-atgtacaagttgcaaatgctgatttgacagaggctaaagccgcattgacagcagcaggtgttaccggcacagcatctgttgttaagatgtcttatactgataataacggtaaaactattgatggtggtttagcagttaaggtaggcgatgattactattctgcaactcaaaataaagatggttccataagtattgatactacgaaatacactgcagatgacggtacatccaaaactgcactaaacaaactgggtggcgccgacggcaaaaccgaagttgtttctattggtggtaaaacttacgctgcaagtaaagccgaaggtcacaactttaaagcacagcctgatctggcggaagcggctgctacaaccaccgaaaacccgctgcagaaaattgatgctgctttggcacaggttgacacgttacgttctgacctgggtgcggtacagaaccgtttcaactccgctattaccaacctgggcaacaccgtaaacaacctgaattctgcccgtagccgtatcgaagattccgactacgcgacctagtagctcgaga-3') was synthesized (and used to replace the 3' end of both the pFliC(-gly) and pcFliC(-gly) constructs after digestion by *Bsr*G I/*Xho* I to create pFliC(-gly)Δ34 and pcFliC(-gly)Δ34.

pFliC(-gly), pcFliC(-gly), pFliC(-gly)Δ34, or pcFliC(-gly)Δ34 were transiently transfected into 293T cells and 2 days later cell lysates were prepared as described [5], total protein concentration was determined by BCA assay (Pierce Thermo Scientific, Walthman, MA, USA), normalized, and subjected to SDS-PAGE (NuPAGE, Invitrogen Life Technologies, Stockholm, Sweden) followed by western blot analysis (anti-HA tag, Covance Research Products, Brussels, Belgium).

The ORF of FliC(-gly) gene and three variants were excised using MfeI and XhoI and inserted into the retroviral expression vector pMSCV-IRES-GFP/neo digested with *Eco*R I and *Xho* I. Constructs were transfected into 293T cells and proteins from cell lysates and supernatants were analyzed for the presence and correct molecular weight of FliC(-gly) and variants by Western blotting as described above (data not shown).

Plasmid vectors expressing gp160 and p24gag from HIV-1 clade B strain Ba-L have been described previously [11].

### 2.2. *In Vitro* Macrophage Stimulations and Retroviral Lethality Screen

All plasmid DNAs were prepared using a Qiagen EndoFree Plasmid Maxi Kit (Qiagen, Hamburg, Germany). Macrophage stimulations were performed as follows. Alveolar macrophages were harvested by BAL from C57BL6/N or TLR5^−/−^ mice backcrossed >10 generations onto C57BL6. Cells were seeded 50,000 cells/well in 96 well plates (Costar) in 50 µL RPMI 1640, 10% heat-inactivated FCS, 2 mM L-glutamine, 100 U/mL penicillin and 100 µg/mL streptomycin (Pierce Thermo Scientific, Walthman, MA, USA) and allowed to settle for 1 h. 50 µL of two day culture supernatants from 293T cells, transiently transfected with 0.8 µg of pFliC(-gly), pFliC(-gly)Δ34, empty vector, recombinant FliC at 100 ng/mL (a gift from A. Gewirtz, Emory, GA, USA) or ultra-pure LPS at 100 ng/mL (InVivogen, San Diego, CA, USA.), was placed on cells and incubated at 37 °C at 10% CO_2_ for 4 h. Cell supernatants were harvested and subjected to standard ELISA to detect secreted mouse TNFα (BioLegend, San Diego, CA, USA).

Retroviral Lethality Screen was performed as follows. pMSCV-IRES-GFP/neo alone or containing FliC(-gly), cFliC(-gly), FliC(-gly)Δ34, or cFliC(-gly)Δ34 ORF were packaged in Phoenix amphotropic virus packaging cells. After transfection media was replaced at day one and viral supernatants were harvested at days two and three. Pooled supernatants were 0.45 µm filtered, concentrated by centrifugation at 2,300 × *g* for 18 h at +4 °C, and frozen at −80 °C. 2 × 10^5^ J2-virus immortalized mouse bone-marrow derived macrophage cells (BcgR) or 293T cells were pre-treated with BX795 (5 µM) for 30 min at 37 °C to improve transduction efficiency [12] followed by 200 µL virus mixed with polybrene 8 µg/mL (Sigma, St. Louis, MO, USA) and centrifuged for 45 min at 27 °C. 4 days after triplicate transductions cells were subjected to analysis for GFP by flow cytometry using a 4-laser LSRII-Fortessa with standard filter sets (BD Bioscience, Stockholm, Sweden). >40,000 non-debris singlets were analyzed in every sample. FACS data was analyzed using FlowJo v9.2 (Tree Star, Ashland, OR, USA).

### 2.3. Mice and Vaccinations

For experiments using OVA antigen female C57BL6/J-crl sub-strain mice (8–12 weeks at priming) from Charles River Laboratories (Sulzfeld, Germany) were used and housed under standard specific pathogen-free conditions (Swedish Institute for Infectious Disease Control). All procedures were reviewed, approved, and performed under both institutional and national guidelines. Plasmid DNA was prepared using a Qiagen EndoFree Plasmid Maxi Kit (Qiagen, Hamburg, Germany) as described by the manufacturer without exception. Vaccinations were done in the animal facility at approximately 24 °C and 60% relative humidity. Mice receiving intramuscular vaccinations were injected with DNA resuspended in PBS in a total volume of 50 μL in one quadricep. Standard ballistic dermal vaccinations were performed as described [5]. For intra-nasal vaccinations, plasmid DNA was resuspended in 0.1 M Tris-HCL, pH 8.0 and placed on ice and mixed with a 1:1 volume of 2% Eurocine cationic N3 lipid (called N3) to make a final volume of 1% N3 lipid adjuvant (see Table 1 for details). Preparation of N3 was carried out as described [13] and was gently stirred with DNA on ice until homogenous then brought to room temperature. Mice were briefly anesthetized with Isofluran, placed dorsal side up, and 4 μL/nostril of N3/DNA mixture was applied to each nostril using a standard laboratory pipette. Mice were gently supported in this position until the mouse revived and attempted to turn over.

**Table 1:** Vaccinated groups for ovalbumin (OVA) experiments.

Group	*n*	Total Immunizations	Route ^a^	ImmunogenpOVA	Adjuvant pFliC(-gly)	Empty Vector pcDNA3.1/Zeo(+)	N3 Lipid
1	7	2	g.g.	0.5 µg	-	0.5 µg	-
2	7	2	g.g.	0.5 µg	0.1 µg	0.4 µg	-
3	8	2	g.g.	0.5 µg	0.2 µg	0.3 µg	-
4	8	2	g.g.	0.5 µg	0.5 µg	-	-
5	7	2	i.m.	10 µg	-	10 µg	-
6	8	2	i.m.	10 µg	2 µg	8 µg	-
7	8	2	i.m.	10 µg	5 µg	5 µg	-
8	8	2	i.m.	10 µg	10 µg	-	-
9	8	2	i.na.	4 µg	-	4 µg	-
10	7	2	i.na.	4 µg	-	4 µg	1%
11	8	2	i.na.	4 µg	1 µg	3 µg	1%
12	8	2	i.na.	4 µg	2 µg	2 µg	1%
13	7	2	i.na.	4 µg	4 µg	-	1%

^a^ g.g.=gene-gun, i.m.=intra-muscular, i.na.=intra-nasal.

For experiments involving gp160/p24gag, eight to ten-week-old female BALB/c mice were purchased from Scanbur BK, Sollentuna, Sweden. Six groups (*n* = 35) were vaccinated with DNA-plasmids expressing gp160 and p24gag (promoter CMV-IE) as previously described [11] with or without adjuvant (Table 2). For plasmid-DNA priming, mice were given 10 µg/plasmid dose/mouse as 5 µL/nostril/mouse with N3 prepared as described above. HIV-1 recombinant protein-boost antigens were gp160 and p24gag (Protein Sciences Inc., Meriden, CT, USA), containing HIV-1 gp160 LAI and p24gag prepared with anionic L3B as described [14]. Mice were given 5 µL of vaccine in each nostril, corresponding to 1 µg recombinant protein antigen and 0%, or 2% of L3B adjuvant or 1% N3 with 5 µg pFliC(-gly) (see Table 2 for details). For delivery of intranasal vaccinations, the mice were treated as above. Groups studied for longetivity of immune responses were immunized three times at three weeks intervals then mice were sacrificed 4, 8, 12, 24, and 36 weeks after the last immunization for analysis. Mice studied for general immune reactivity were sacrificed 4 weeks after the final immunization.

**Table 2:** Vaccinated groups for gp160/p24gag experiments.

Group	*n*	Total Immunizations	Priming			Boosting	
			ImmunogenP ^a^	Adjuvant	EmptyVectorpcDNA3.1/Zeo(+)	ImmunogenB ^b^	Adjuvant
1	35	3	pgp160(5 µg) pGagp24(5 µg)	None	-	rgp160/rp24gag (1 µg each)	None
2	35	3	pgp160(5 µg) pGagp24(5 µg)	N3 (1%)	-	rgp160/rp24gag (1 µg each)	L3B(2%)
3	35	3	pgp160(5 µg) pGagp24(5 µg)	pFliC(-gly) (5 µg)	-	rgp160/rp24gag (1 µg each)	L3B(2%)
4	35	3	pgp160(5 µg) pGagp24( 5µg)	N3(1%) + pFliC(gly) (5 µg)	-	rgp160/rp24gag (1 µg each)	N3(1%) + pFliC(gly) (5 µg)
5	35	3	pgp160(5 µg) pGagp24(5 µg)	None	5 µg	rgp160/rp24gag (1 µg each)	N3(1%) + pcDNA3.1 (5 µg)
6	35	3	pgp160(5 µg) pGagp24(5 µg)	N3(1%)	5 µg	rgp160/rp24gag (1 µg each)	N3(1%) + pcDNA3.1 (5 µg)
Saline	30	3	Saline				

^a^ Plasmid DNA; ^b^ Recombinant protein.

### 2.4. Antibody, Mucosal Cytokines and T Cell Analysis

Anti-OVA humoral responses were performed as follows. Briefly, serum, fecal pellets (100 mg feces solublized in 1 mL PBS with protease inhibitors, Complete Mini, Roche, ) and vaginal washings (50 μL of PBS with protease inhibitors, as above) were subjected to anti-OVA ELISA as described [5]. Assessment of antigen-specific IgA in lungs was done as follows. Isolated lungs were rinsed in cold PBS then minced in PBS with protease inhibitors. Solids were removed by centrifugation and total IgA in washings were determined by ELISA, using a primary monoclonal goat-anti mouse IgA (Sigma 098K4823 clone ISO2-1KT, St. Louis, MO, USA) and secondary rabbit anti-goat IgG HRP (Dako, Stockholm, Sweden). IgA anti-OVA titers were determined by ELISA then normalized for total IgA content. Individual samples were tested in triplicate at a dilution of 1/10. Amino acids were numbered according to the Los Alamos Data Base on Retroviruses (peptide source: AIDS Research and Reference Reagent Program, Division of AIDS, NIAID, NIH: from DAIDS, NIAID and J&J, San Diego, CA, USA) [15]. For IgG anti-gp160 measurements, individual mouse sera were diluted in ten-fold steps from a starting dilution of 1:100 in ELISA-buffer (2.5% dry milk and 0.05% Tween-20 (Sigma, St. Louis, MO, USA) in PBS to end-point. Goat-anti-mouse IgG (H+L)-HRP secondary conjugate (Bio-Rad, Hercules, CA, USA) was used, diluted 1:3,000 to detect IgG anti-gp160 immune complexes. Anti-gp160 IgA and IgG isotype subclasses were measured using a mouse monoclonal antibody isotyping reagent (Sigma, St. Louis, MO, USA) according to the manufacturer’s protocol with peroxidase-conjugated anti-Goat IgG (Sigma, St. Louis, MO, USA), diluted 1:2,000. For developing ELISA reactions, O-phenylenediaminedihydrochloride (OPD) (Sigma, St. Louis, MO, USA) was used. Based on earlier studies, an OD of 0.2 was set as the cut-off value for positive samples. Clade A, Uganda 29 (UG29) and C, Brazil (BR25) envelope antigens were obtained from the AIDS Research and Reference Reagent Program, Division of AIDS, National Institute of Allergy and Infectious Diseases, National Institutes of Health (NIAID, NIH, Bethesda, MD, USA). Mucosal wash IgA analyses were performed as previously described [16,17,18]. Briefly, IgA was isolated from secretions collected by nasal washing using Kaptive/IgA/IgE reagents (Biotech IgG, Copenhagen, Denmark) as recommended by the manufacturer. IgA quantities were determined using an in-house murine IgA capture ELISA, and commercial murine IgA (1 mg/mL, Sigma, St. Louis, MO, USA) was used to prepare a standard curve. The purified IgA and the standard IgA were diluted in ten-fold serial dilutions. From each dilution, 100 µL was added to each well of a 96-microwell plate pre-coated with rabbit anti-murine IgA (Dakopatts AB, Sollentuna, Sweden). Goat-anti-mouse IgA-HRP secondary conjugate (SouthernBiotech, Birmingham, AL, USA), diluted by 1:3,000, was used to detect IgA anti-gp160. The total amounts of IgA in nasal samples were determined by comparing the OD values of the test samples with the IgA standard and final anti-gp160 values were normalized to total IgA values.

T-cell responses to OVA was performed as described [5]. Briefly, spleens were isolated, single-cell suspensions were Ficoll-purified, washed twice with PBS and used in IFNγ ELISPOT analysis according to the manufacturer guidelines (Mabtech, Nacka, Sweden). Ag restimulation was performed using either the H-2Kb binding OVA peptide SIINFEKL (257-264) at 1 μM final concentration or the I-Ab binding OVA peptide ISQAVHAAHAEINEAGR (323-339) at 1 μM final concentration (GenScript, Piscataway, NJ, USA).). Cell reactivity was confirmed by incubation with ConA. Spot-forming cells were quantified after 24 h incubation and counted by AID ELISPOT reader (AutoImmun Diagnostika, Straßberg, Germany).

T cell immune responses to gp160 were measured using a cell-in-well murine cytokine capture-ELISA assay as described previously [11]. Briefly, 96-well ELISA plates were coated with capture anti-IFNγ (AN18) or anti-IL-5 (TRFK4) according to the manufacturer’s protocol (Mabtech, Nacka, Sweden) overnight at 4 °C. Following well washing and blocking according to the manufacturer’s protocol 2.5 × 10^5^ ficoll-purified splenocytes from individual mice were added to each well, either with or without recombinant HIV-1 gp160, p24gag (Protein Sciences Inc., Meriden, CT, USA), control antigen (Sf9 cell lysate), the positive control Concanavalin A (2.5 µg/mL, Sigma, St. Louis, MO, USA) or RPMI 1640-medium alone. Plates were incubated at 37 °C, 5% CO_2_ for 5–6 days. Cells were then removed, plates were washed with PBS, and biotinylated detection antibodies were added, washed, followed by streptavidin-ALP (Mabtech, Nacka, Sweden). The plates were developed with substrate solution (Mabtech, Nacka, Sweden) for 5–10 min until spots became visible, and the color reaction was stopped by 2.5 M H_2_SO_4_. Plates were then read in an ELISA reader (BioRad, Hercules, CA, USA) at 405 nm.

T-cell proliferation to gp160 was performed as described previously [18] using 1 µg/mL of rgp160 or rGag p24 as specific antigens.

Nasal washings were performed trice with 25 µL PBS/nostril on Isofluran sedated mice kept in supine position. Collected washings were frozen until analyzed. Nasal washings were tested individually for the cytokines IL-6, IFNα, and IFNγ according to the manufacturer’s protocols (R&D Systems, Minneapolis, MN, USA).

### 2.5. HIV-1 Neutralization Assay

The HIV-1 neutralization assay was performed as described previously [11]. The viral isolates used for the neutralization were the subtype B laboratory strains IIIB LAI (vaccine homologus) and the primary subtype B isolate 6,794. Briefly, the sera from mice were pooled group wise and inactivated at 56 °C for 1 h to prevent complement-mediated neutralization. Sera were diluted in RPMI 1640 medium (Invitrogen Life Technologies, Stockholm, Sweden) in 96-well tissue culture plates (Nunc microwell plates, Nunc, Pierce Thermo Scientific, Walthman, MA, USA). Dilutions were mixed with virus and incubated at 37 °C for 1 h followed by the addition of 1 × 10^5^ human PBMCs (activated by phytohemagglutinine and rIL-2; PeproTech, Rocky Hill, NJ, USA) or Jurkat T cells. The cultures were incubated at 37 °C in 5% CO_2_ over night, after which the cells were washed twice with RPMI 1640. After 6 days of culture, the presence of HIV-1 p24 antigen in the culture medium was measured by ELISA [19]. The background in the p24 ELISA was determined for each plate and subtracted from all wells before the percentage neutralization was determined as [1-(mean p24 OD in the presence of test serum/mean p24 OD in the absence of test serum)] × 100. Ethical permission for use of huPBMCs was approved by the ethical committee at Linköping University Hospital.

Statistical analysis was performed using GraphPad Prism 5 (La Jolla, CA, USA). Comparisons between groups with the HIV-1 antigens were performed by using the non-parametric Mann-Whitney U test with Bonferroni correction, *p* < 0.05 was considered significant. 

## 3. Results and Discussion

### 3.1. Construction of Secreted FliC Adjuvant

**Figure 1:**
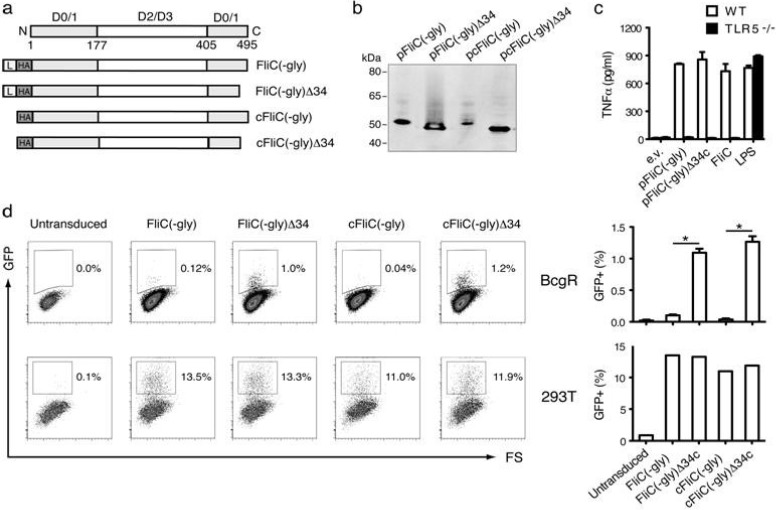
Flagellin (FliC)(-gly) variants for *in vivo* and *in vitro* use activate innate immune responses. (**a**) Depiction of pFliC(-gly) and variants relative to FliC polypeptide and domains produced by *S. typhimurium*. Grey domains D0/1 indicate conserved regions important for activating innate immune responses. L and HA indicates a leader and HA-epitope tag domains respectively. Names of the four FliC(-gly) constructs used in this study are indicated to the right of the drawings; (**b**) Western blot analysis of cellular lysates from 293T cells transfected with the indicated constructs. Apparent molecular weights were determined by comparison to the standard depicted to the left of the blot. Signals were not detected from cells transfected with empty vector (data not shown); (**c**) Release of TNFα from B6 alveolar macrophages but not Toll-like Receptor 5 (TLR5) −/− alveolar macrophages after stimulation with FliC(-gly) and FliC(-gly)Δ34. Supernatant from 293T cells transfected with pFliC(-gly) and pFliC(-gly)Δ34 vectors was incubated with cells for 4 h followed by analysis of secreted TNFα by ELISA. Data are mean ± SEM of triplicate samples representative of two independent experiments; (**d**) Activation of pyroptotic cell death by retroviral transduction of BcgR macrophages with constructs expressing FliC(-gly) but not FliC(-gly)Δ34 as determined by GFP expression. Upper panels represent representative data from BcgR cells transduced with FliC(-gly), FliC(-gly)Δ34 and controls (as indicated) when comparing GFP and forward-scatter (FS) parameters. Quantitative data of the percentage of GFP positive BcgR cells from each construct after transduction. Lower panel represent representative data from 293T cells transduced in identical fashion. Data are mean ± SEM of GFP positive cells observed during three independent transduction experiments. * Differences of the response relative to the FliC(-gly) construct without Δ34 defined as *p* ≤ 0.05 calculated using a two-tailed unpaired Student *t* test.

A secreted variant of flagellin with reduced glycosylation (called pFliC(-gly)), based on the pFliC-Tm(-gly) plasmid [5], was constructed by removing the human transmembrane PDGFR domain from the ORF to eliminate potential immune responses to this region and to prepare a base vector for adjuvant use. Three additional variants of pFliC(-gly) were also constructed to test the ability of FliC(-gly) to activate the two known innate immune receptors capable of sensing flagellin TLR5 and NLRC4/Naip5. These four constructs are depicted in Figure 1a relative to the defined domains of *Salmonella typhimurium* FliC. To prepare pFliC(-gly) control variants capable of activating cytoplasmically expressed NLRC4/Naip5 we recloned the FliC(-gly) insert sans leader sequence (pcFliC(-gly)). We also prepared additional control versions of pFliC(-gly) and pcFliC(-gly) removing the COOH-34 amino-acids of FliC(-gly) shown to activate NLRC4/Naip5 [20]. These versions were designated pFliC(-gly)Δ34 and pcFliC(-gly)Δ34 respectively. All four vectors were capable of expressing proteins of predicted size with an apparent polypeptide of approximately 52 kDa for pFliC(-gly) and pcFliC(-gly) and approximately 48 kDa for pFliC(-gly)Δ34 and pcFliC(-gly)Δ34 (Figure 1b). To determine if secreted FliC(-gly) protein produced from pFliC vectors could activate TLR5 culture supernatants from pFliC(-gly), pFliC(-gly)Δ34 transfected 293 cells, or recombinant FliC protein were applied to alveolar macrophages from B6 or TLR5-deficient mice. Plasmid vectors produced full-length or Δ34 secreted FliC(-gly) able to activate B6 alveolar macrophages to produce TNFα but not macrophages from TLR5-deficient mice (Figure 1c). To determine if secreted FliC(-gly) has the potential to activate cytoplasmic NLRC4/Naip5 inflammasome responses we performed a retroviral lethality screen using the macrophage cell line BcgR which undergoes pyroptosis in the presence of the COOH-terminal tail of FliC [21]. This assay detects the ability of macrophages virally transduced with genes expressing GFP as well as various flagellin constructs to undergo pyroptotic cell death in response to whole flagellin dependent on the NLRC4/Naip5 35 amino-acid carboxy-terminal activating domain [20,21]. GFP positive BcgR cells are taken as evidence of a lack of NLRC4/Naip5 activation while GFP negative cells, relative to GFP positive identically transduced 293T control cells, are taken as evidence of NLRC4/Naip5 activation. FliC(-gly), cytoplasmic expressed FliC (cFLiC(-gly)), and a variant of each lacking the final 34 amino-acid COOH-tail (Δ34) (Figure 1a) were subcloned into the retroviral vector pMSCV-IRES-GFP which are designed to produce FliC(-gly) and variants as well as GFP upon transduction. Various FliC(-gly) constructs were Amphotropic packaged, and used to transduce BcgR or 293T cells. Using GFP as a reporter for FliC expression we observed that all versions of FliC(-gly) were expressed at nearly equal frequency in 293T cells indicating that all vectors were packaged with equal efficiency and could deliver GFP and FliC genes (Figure 1d). However, when identical vector preparations expressing FliC(-gly) were trandsuced into BcgR cells we observed GFP expression only with Δ34 versions (Figure 1d). These results demonstrate that secreted form of FliC(-gly) we use as an *in vivo* adjuvant has the ability to activate NLRC4/Naip5 pyroptotic cell death when expressed in a responsive cell type. 

FliC(-gly) produced from pFliC(-gly) stimulated TNFα production in a TLR5-dependent manner as well as inflammatory cell death (pyroptosis) dependent on a defined FliC region known to be a NLRC4/Naip5 agonist. These results suggest that TLR5 and NLRC4 expressed *in vivo* could be important factors in the adjuvant effects of pFliC(-gly) in immunized mice. It is interesting that FliC(-gly) destined for secretion has the capacity to activate the cytoplasmic flagellin detectors NLRC4/Naip5. We consider it likely that a portion of secreted FliC(-gly) undergoing translation is retro-translocated from the endoplasmic reticulum back into the cytoplasm where it may detected by NLRC4/Naip5 leading to the induction of pyroptosis.

### 3.2. pOVA DNA Vaccinations; Timeline, Routes, and Dose

**Figure 2:**
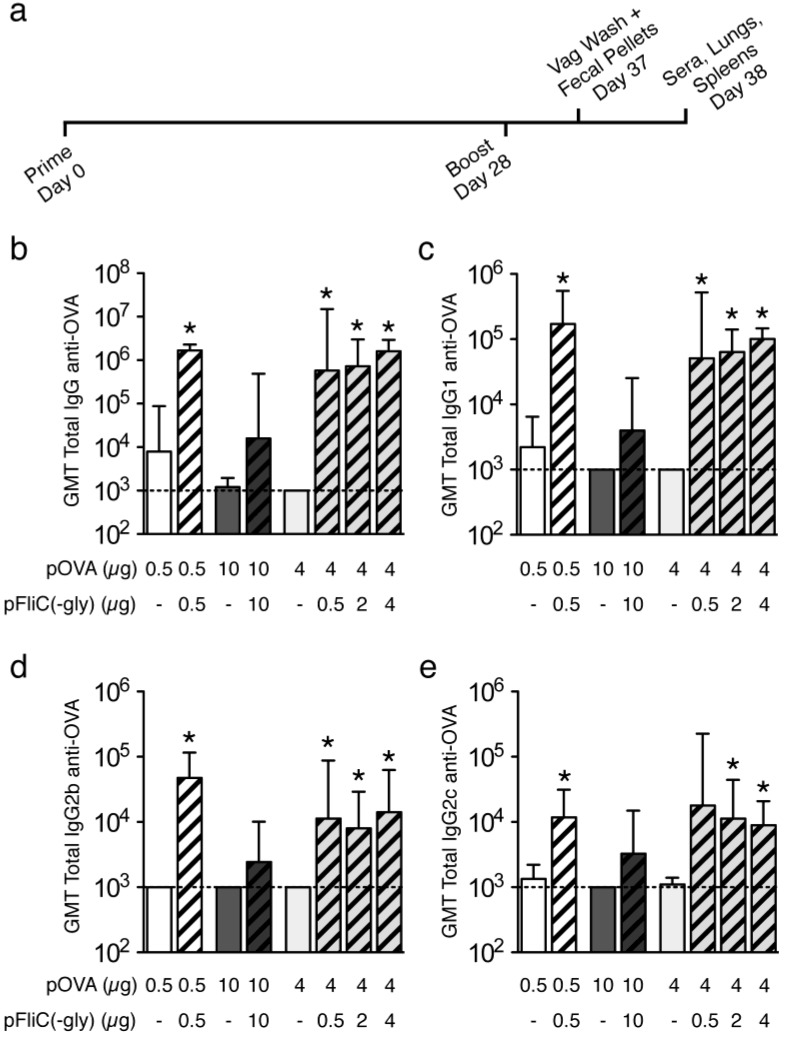
Vaccination schedule and serum antibody responses to OVA. (**a**) Immunization and sample isolation timeline; (**b**) Anti-OVA total IgG responses. Anti-OVA IgG1 (**c**), IgG2b (**d**), IgG2c (**e**) responses. (White bars) g.g. (Dark Grey Bars) i.m. (Grey bars) i.na. immunized mice. Striped bars indicate the use of pFliC(-gly). Results are representative of two independent experiments (*n* = 7–8 mice/group). The concentration of OVA-specific Abs are expressed as the reciprocal of the last dilution of samples giving an OD equal to, or higher than, the mean + 3 SDs (the determined cutoff value for the assay) of the values of serum samples from unimmunized mice. Absorbance values equal to or above the cutoff value were considered positive. The error bars represent 95% confidence intervals calculated from the geometric mean titers. * Differences of the response relative to pOVA immunizations without pFliC(-gly) defined as *p* ≤ 0.05 were considered significant using a two-tailed unpaired Student *t* test.

To compare the effectiveness of secreted flagellin (pFliC(-gly)) as a DNA adjuvant by various routes, DNA vaccinations were carried out using plasmid pOVA together with empty vector (pcDNA3.1/Zeo(+)) or with vector expressing pFliC(-gly). Empty vector control was used to include possible adjuvant effects contributed by sensing of B-DNA by innate immune receptors [22,23] but to exclude adjuvant effects contributed by secreted flagellin (Table 1). Vaccinated mice were primed once, boosted once and then sampled 9 and 10 days later (Figure 2a). The amount of total DNA given to the mice varied and was dependent on the limitations of the delivery route (Table 1). Mice were vaccinated by three different routes representative of dermal (gene-gun, g.g.), systemic (intramuscular, i.m.), and mucosal tissues (intra-nasal, i.na.). A constant sub-optimal amount of pOVA was used with each route (0.5 μg/g.g., 10 μg/i.m., 5 μg/i.na.) to allow the study of the adjuvant effects of flagellin.

### 3.3. Antibody Immune Responses to pOVA DNA Vaccination

To determine if pFliC(-gly) promotes humoral immune response to co-delivered DNA-encoded antigen (pOVA), we studied antigen-specific antibody responses in dermal, systemic, and mucosal compartments. When anti-OVA antibody responses were examined in the sera of vaccinated mice we observed that the pFliC(-gly) adjuvant increased the antigen-specific total IgG antibodies in the sera of mice vaccinated with pOVA by all three routes (Figure 2b). These responses were dependent on the dose of pFliC(-gly) used as mice given 0.1 or 0.2 μg of pFliC(-gly) by g.g. or mice given 2 or 5 μg of pFliC(-gly) by i.m. did not exhibit any increases in anti-OVA antibody responses (data not shown; Table 1, Groups 2, 3 and 6, 7 respectively). Significant increases were seen when mice were vaccinated by g.g. or i.na., however, we also observed a reproducible trend of pFliC(-gly) to promote increases in anti-OVA total IgG when mice were vaccinated i.m. To see if the adjuvant effects of pFliC(-gly) delivered by various routes affected skewing of anti-OVA IgG isotypes, we analyzed the titers of anti-OVA IgG1, IgG2b, and IgG2c in the sera of vaccinated mice. We observed increases in all three IgG isotypes when pFliC(-gly) adjuvant was used, regardless of the route of delivery (Figure 2c–e).

**Figure 3:**
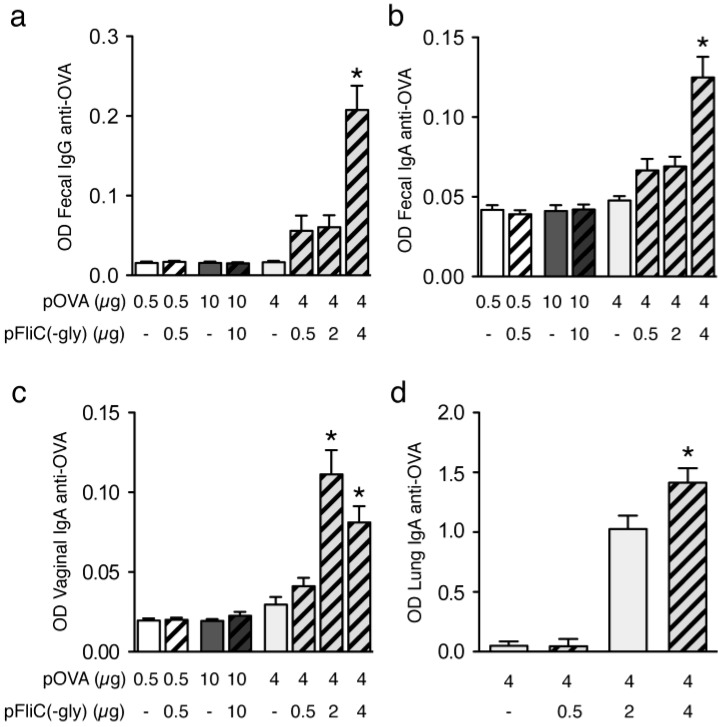
Mucosal antibody responses to OVA. (**a**) Fecal anti-OVA IgG and (**b**) IgA responses; (**c**) Vaginal anti-OVA IgA responses. (White bars) g.g. (Dark Grey Bars) i.m. (Grey bars) i.na. immunized mice. Striped bars indicate the use of pFliC(-gly); (**d**) Lung anti-OVA IgA responses shown are from mice only vaccinated i.na. and immunizations given are shown below the axis. Results are representative of two independent experiments (*n* = 7–8 mice/group). The concentration of OVA-specific Abs in samples are expressed as OD equal to, or higher than, the mean OD of the values of samples from unimmunized mice. The error bars represent SEM calculated from the mean OD. * Differences of the response relative to pOVA immunizations without pFliC(-gly) defined as *p* ≤ 0.05 were considered significant using a two-tailed unpaired Student *t* test.

To assess whether DNA vaccination of mice with pOVA and empty vector or pFliC(-gly) by various routes could elicit antibody responses in mucosal compartments, we collected extracts from fecal pellets, vaginal washes, and extracts from lung homogenates. We observed no significant differences in total amounts of total IgG or IgA immunoglobulins isolated from g.g., i.m. or i.na.-immunized animals (data not shown). Fecal extract samples were assessed for the relative amount of anti-OVA total IgG and IgA. We were able to detect significant increases in fecal anti-OVA IgG and IgA in the groups of mice vaccinated intranasally with pOVA together with the highest amounts of pFliC(-gly), but not when the same plasmids were delivered by g.g. or i.m. (Figure 3a–b). Similarly, only animals receiving the highest doses of pFliC(-gly) and pOVA intranasally developed measurable anti-OVA IgA in the vaginal washes (Figure 3c) and lungs (Figure 3d).

We find it interesting that pFliC(-gly) promotes antigen-specific IgG and IgA responses to in mucosal compartments after mucosal delivery but not when it is delivered systemically or dermally. Despite this specificity we observed antigen-specific IgG in the sera by all routes. Other studies using purified flagellin protein and mucosal cell populations however, has revealed that the small intestine lamina propria contains CD103^+^ dendritic cells which express TLR5 and respond directly to flagellin to promote T cell-independent class switching of naive B cells from IgM^+^IgD^+^ to IgA [24]. It may be that a similar phenomenon occurs *in vivo* when flagellin is present in the compartments of the nasal mucosa and upper airway. However, it is not known why flagellin acts to promote humoral immune responses by all routes explored, but does not elicit mucosal antibodies when delivered systemically or dermally. Differences in the numbers, types, or the immune-skewing potential of flagellin-responsive cells interacting with flagellin after i.m. or g.g. delivery could be responsible for these effects.

### 3.4. Cellular Immune Responses to pOVA DNA Vaccination

**Figure 4:**
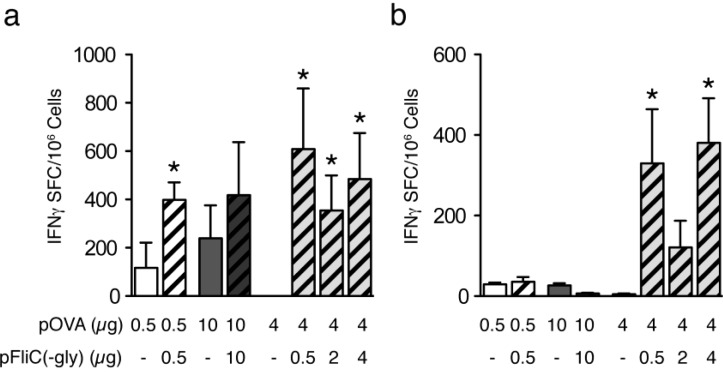
Class I- and Class II-dependent T cell responses to OVA. IFNγ ELISPOT analysis of splenic T cell responses to (**a**) Class-I and (**b**) Class-II MHC binding OVA peptides after vaccination. (White bars) g.g. (Dark Grey Bars) i.m. (Grey bars) i.na. immunized mice. Striped bars indicate the use of pFliC(-gly). Results are representative of two independent experiments (*n* = 7–8 mice/group). Data is expressed as the calculated geometric mean of the Ag-stimulated cells minus unstimulated cells. The error bars representSEM calculated from the mean SFC/10^6^ splenocytes. Statistical analyses were conducted using a two-tailed Student *t* test. * Differences of the response relative to pOVA immunizations without pFliC(-gly) defined as *p* ≤ 0.05 were considered significant using an two-tailed unpaired student *t* test.

MHC class I-dependent responses were analyzed by stimulation of splenocytes from immunized mice with peptide representing the immunodominant OVA H2-Kb restricted epitope. We observed significant increases in the numbers of antigen-specific IFNγ-producing cells in mice receiving either g.g. or i.na. immunization with pOVA and pFliC(-gly) when compared to mice receiving pOVA together with empty vector (Figure 4a). We also observed a reproducible trend of pFliC(-gly) to promote antigen-specific increases in IFNγ-producing cells when mice were vaccinated i.m. (Figure 4a). These Class I cellular responses were dependent on the dose of pFliC(-gly) delivered as mice given 0.1 or 0.2 μg of pFliC(-gly) by g.g or mice given 2 or 5 μg of pFliC(-gly) i.m. did not exhibit detectable Class I-dependent responses (data not shown; Table 1, Groups 2, 3 and 6, 7 respectively). When Class II-dependent cellular immune responses were studied by stimulating splenocytes with the immunodominant I-Ab binding OVA peptide we observed significant increases in the numbers of antigen-specific IFNγ-producing cells in mice receiving pOVA intranasally together with the highest amounts of pFliC(-gly), but not with pOVA and empty vector (Figure 4b). We did not observe any OVA-specific class II-restricted responses after i.m or g.g. immunization (Figure 4b).

We observed unique Class-II dependent IFNγ-responses in the spleen after mucosal delivery of pFliC(-gly) but not when delivered systemically or dermally. This mirrors our observations of mucosal IgG and IgA with pFliC(-gly) use. Why does pFliC(-gly) promote strong Th1-like CD4^+^ T cell and IFNγ-producing CD8^+^ T cell responses when delivered mucosally but only promotes increases in IFNγ-producing CD8^+^ T cells when applied systemically or dermally? It could be that splenic Th1-like CD4^+^ cells have trafficked to other locations before analysis or are below the level of detection. Alternatively, there may be a strong ability of FliC to promote CD4+ responses when delivered mucosally. It has been observed that certain mucosal DC populations expressing TLR5 have a special ability to promote flagellin-specific CD4^+^ T cell responses [25]. However, it remains to be seen if these TLR5-dependent responses can be extended to other antigens encountered in the same environment as flagellin. Nevertheless, as an adjuvant, we find it interesting that flagellin can promote different immune responses to the same antigen encountered in different environments. This may have relevance to immune responses elicited by flagellated pathogens.

Adjuvant effects of pFliC(-gly) were dose-dependent. Lower doses delivered intranasally re-capitulated the effects seen after systemic and dermal routes giving increases in anti-OVA IgG in the sera as well as IFNγ-producing Class I-dependent cellular responses. Higher doses of pFliC(-gly) however, were able to induce mucosal anti-OVA IgG and IgA responses. These observations suggest there may be a lower threshold for flagellin to promote systemic responses to an antigen compared to mucosal responses, which might require more of the adjuvant. Whether this could be through the triggering of a threshold of pre-existing cells at the vaccination site, recruitment of new cell populations to the site after vaccination, or differences in triggering TLR5 and NLRC4 responses is not known.

### 3.5. gp160 DNA and Protein Vaccinations; Timeline and Antibody Responses

**Figure 5:**
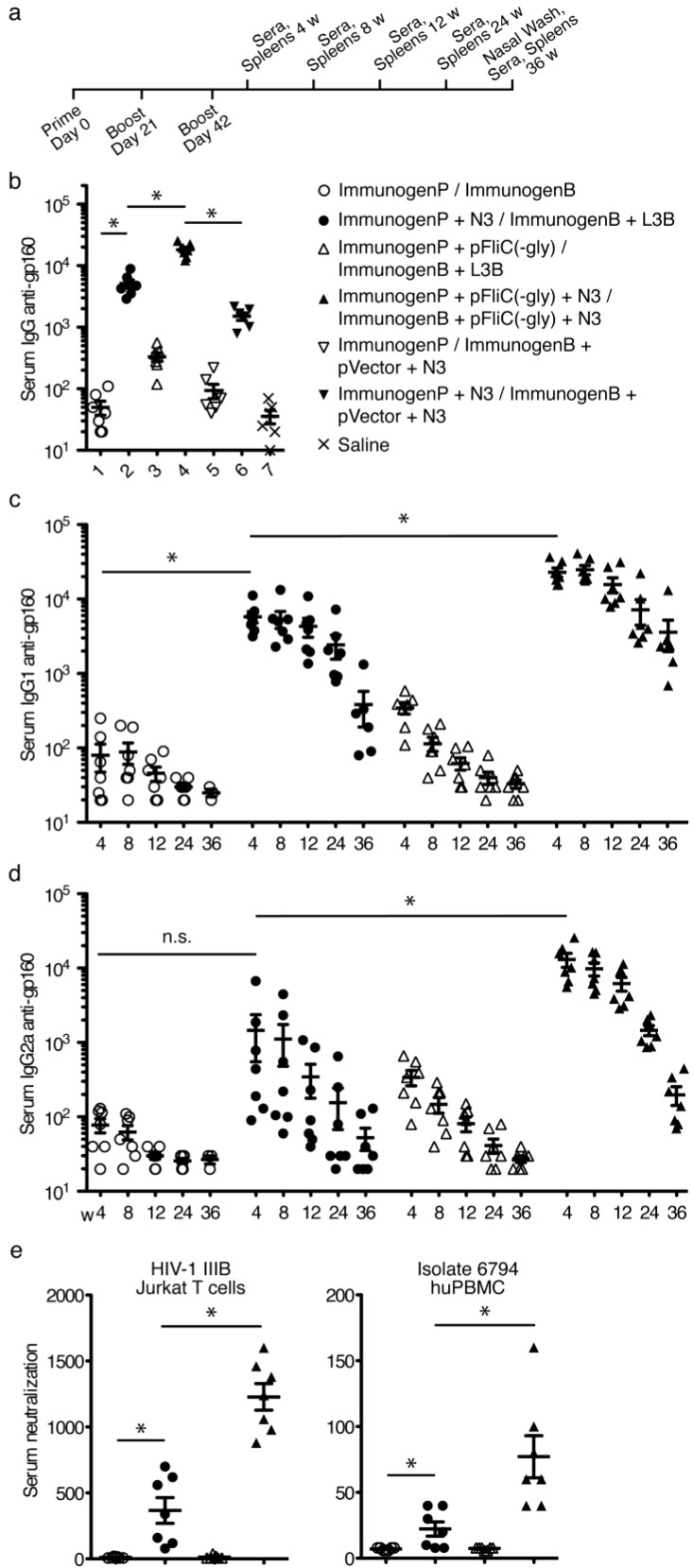
Vaccination schedule, serum antibody responses to gp160, and virus neutralization titers. (**a**) Immunization and sample isolation timeline. Priming (ImmunogenP, plasmids) and boostings (ImmunogenB, rec proteins) are indicated in days while time after the final boost are indicated in weeks. Immunization details are listed in Table 2; (**b**) Serum IgG titer against rgp160 at 4 weeks post immunization in all seven study groups; (**c**) Serum titer anti-rgp160IgG1 isotype kinetics in the four first study groups in Table 2; (**d**) Serum end-point titer anti-rgp160 IgG2a isotype kinetics in the four first study groups. The concentration of rgp160-specific Abs are expressed as the end-point titers giving an OD equal to, or higher than, the mean + 3 SDs (the determined cutoff value for the assay) of the values of serum samples from unimmunized mice. Absorbance values equal to or above the cutoff value were considered positive; (**e**) Serum neutralization of HIV shown as IC50 in serum samples of the four first study groups in Table 2. The TCID50 (the reciprocal of the virus dilution where 50% of the cultures were infected) of IIIB (LAI) or 6794 was incubated with sample mouse serum (dilutions: 20, 60, 180, 540, 1 620). 5 × 10^4^ cells well were then added, incubated, washed, and incubated for 7 days. Culture supernatants were tested for virus production by HIV-1 p24 capture ELISA. The lowest serum concentration giving a 50% reduction (IC50) of ELISA absorbance value compared with the mean of the negative controls are presented [19]. Statistical analyses were conducted using a two-tailed unpaired Student *t* test. * Differences of the responses between compared groups defined as *p* ≤ 0.05 were considered significant. n.s. = non-significant. Comparisons between groups with the HIV-1 antigens were performed by using the non-parametric Mann-Whitney U test with Bonferroni correction, *p* < 0.05 was considered significant.

Experiments with pOVA and pFliC(-gly) indicated that delivery of plasmids using N3 and the intranasal route was able to promote cellular immune responses as well as humoral mucosal immune responses. To compare the effectiveness of secreted flagellin to promote immune responses to a clinical antigen using a heterologus prime/boost regime, priming intranasal DNA vaccinations were carried out using plasmid pgp160Lfai/pRev [16] with delivery lipid N3 alone or together with pFliC(-gly). Boostings were performed using recombinant gp160 proteins with a protein-delivery lipid L3B alone or together with N3 mixed with pFliC(-gly) (Table 2). In these experiments mice were given doses of antigen and adjuvant believed to maximize detectable responses. Mice were primed, boosted, and analyzed according to the indicated timeline (Figure 5a). Four weeks post-final boost serum total-IgG titers anti-rgp160 indicated that addition of N3 to pgp160 was able to strongly promote anti-gp160 antibody responses (Figure 5b). Similar to responses seen using OVA, addition of N3/pFliC(-gly) to the immunization regime enhanced antigen-specific antibody titers further (Figure 5b). The adjuvant effect was dependent on N3. Likely due to it’s ability to encapsulate plasmid DNA and protect it from the degradative environment of the mucosal compartment. These higher titers of antigen-specific IgG in the sera and the presence of antigen-specific mucosal IgA indicate that the mucosal adjuvant effects of pFliC(-gly) are not limited to experimental antigens. 

Kinetic analysis of anti-gp160 IgG isotypes revealed that addition of N3 to pgp160 was able to promote anti-gp160 IgG1 at 4 weeks following the final boost. This titer was generally sustained to 24 weeks but fell sharply by week 36 (Figure 5c). A similar trend was seen when anti-gp160 IgG2a was studied (Figure 5d). When pFliC(-gly) was added to N3/pgp160 vaccinations IgG1 and IgG2a anti-gp160 titers were enhanced further as well as sustained to later time points (Figure 5c,d). These results suggest that the adjuvant effects of pFliC(-gly) not only boost antigen-specific antibody but that these effects may also lead to longer persistence of antibody.

One should bear in mind that the antigen doses used were chosen to be relatively weak at inducing immune responses when given via the nasal route in small volume once or twice without adjuvant. Therefore, it was not surprising to see a short-lived serum and mucosal antibody response unless adjuvant was used. Longevity of total anti-gp160 IgG was significantly enhanced in groups where the N3 adjuvant was used especially when combined with pFliC-DNA. Since the N3 adjuvant has cationic and surfactant properties one proposed mechanism would be that there is an increased mucosa-penetrating property when HIV-antigen and adjuvant is given in mixture. This would increase the amount of antigen reaching below the mucosal surface, thereby making the antigen available at higher dose for antigen-presenting cells in the mucosa. Equally important, the capacity of the serum immunoglobulins to neutralize HIV-1 virus *in vitro*, both cell-line adapted (HIV-1IIIB), and primary patient isolate (HIV-1 6794B) remained clearly detectable at 36 weeks in serum from groups 2 and 4 (Figure 5e). 

**Figure 6:**
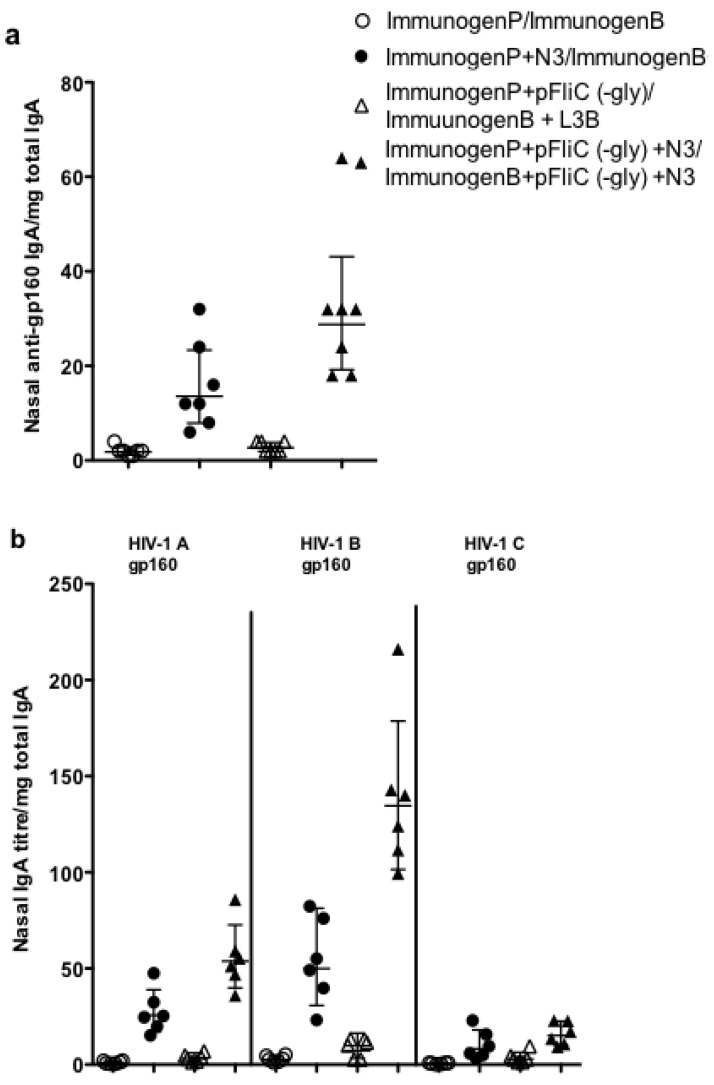
Mucosal antibody responses to gp160. (**a**) Nasal IgA anti-rgp160; (**b**) Nasal IgA anti-gp160 cross-reactivity against clade A, B, and C envelope antigens. Priming (ImmunogenP, plasmids) and boosting (ImmunogenB, rec proteins) groups are shown in the key. Immunization details are listed in Table 2. ELISA was performed using individual serum from the indicated immunization groups. Absorbance values equal to or above the cutoff value were considered positive. Statistical analyses were conducted using a two-tailed unpaired Student *t* test. * Differences of the responses between compared groups defined as *p* ≤ 0.05 were considered significant.

Although we observe enhanced titers of anti-viral spike antigen antibodies in the serum of animals immunized with antigen and pFliC(-gly) adjuvant the antibodies in mucosal secretions may be more likely to potentially neutralize viral particles. Studies of vaginal IgA anti-gp160 responses had assay backgrounds that precluded the determination of antigen-specific titers (data not shown). As a surrogate location representative of antigen-specific IgA responses we studied the titers of IgA anti-gp160 harvested from nasal washes. We observed trends similar to those found in the serum. Addition of N3 to pgp160 vaccinations followed by L3B protein boostings lead to clear IgA anti-gp160 titers which could be further enhanced by the addition of pFliC(-gly) (Figure 6a). The ability of nasal IgA anti-gp160 to cross-react against homologous (clade B) as well as heterologus clades (A and C) of HIV-1 gp160 was also tested. We observed nasal wash reactivity to clades A and B (Figure 6b). As with serum IgG, N3 could promote detectable anti-gp160 antibodies while pFliC(-gly) could enhance responses even further (Figure 6b). These results indicate that higher titers of antigen-specific clade B160 IgA also correlate with higher titers of antibody able to cross react with HIV-1 clade A gp160. Increases in cross-clade reactivity may likely be a behavior central to the development of an effective vaccine.

**Figure 7:**
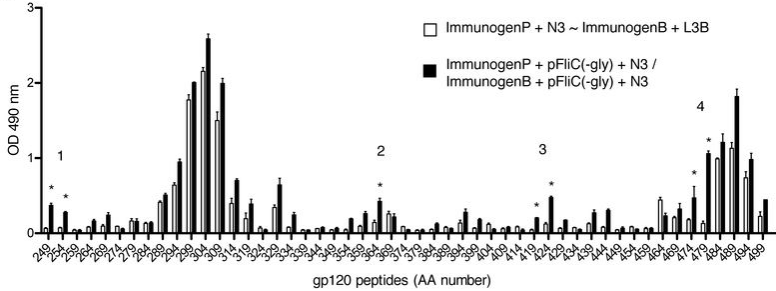
B cell epitope mapping to C2-C5 region of gp160 after immunization with gp160 with and without adjuvant. ELISA was performed using group-pooled serum (equal volumes) from immunization group 2 (*n* = 35) or 4 (*n* = 35) against individual peptides. Priming (ImmunogenP, plasmids) and boosting (ImmunogenB, rec proteins) groups are shown in the key. Immunization details are listed in Table 2. The concentration of gp160-peptide specific Abs are expressed as the end-point titers giving an OD equal to, or higher than, the mean + 3 SDs (the determined cutoff value for the assay) of the values of serum samples from unimmunized mice. Absorbance values equal to or above the cutoff value were considered positive. Statistical analyses were conducted using a two-tailed unpaired Student *t* test. * Differences of the responses between compared groups defined as *p* ≤ 0.05 were considered significant.

To determine the breadth of antibody responses against hypervariable regions of gp160 within antigen-specific IgG we performed B cell epitope mapping of group-pooled serum against individual 20-mers of gp160 from AA249–499 containing the V3 variable loop region. We observed clear populations of IgG anti-gp160 peptide immune responses in the sera of mice immunized with pgp160 with N3 followed by boosting with rgp160 protein with L3B (Figure 7a). However, the addition of pFliC(-gly) to immunizations expanded the number of detectable populations by five (Figure 7a). Analysis of amino acid identities and similarities between FliC and clade B LAI gp160 within the region encoded by the peptides were performed using NCBI BLASTP analysis (v2.2.26+) with default settings. Two regions were identified containing identity and similarity. Of these two regions only one (containing 36% identities and 55% similarity) overlapped with a region of increased reactivity (peptides AA 439 and 444) and was excluded. There were no regions of alignment identified within the 5 annotated populations that exhibited equal or greater identity and similarity than the 22AA region. 

These use of pFliC(-gly) appears to promote a broadening of B cell epitope reactivity to rgp160 and/or presentation of additional “masked” epitopes. Similar responses have been seen in response to a TLR-adjuvanted malaria vaccine using advanced techniques [26]. However, how this increased reactivity occurs is not known. It may be that the higher titers of anti-gp160 elicited by use of pFliC(-gly) revealed reactivity normally below threshold when samples from un-adjuvanted groups were studied. Additionally, it may be that pFliC(-gly) is able to promote expansion of B cell populations normally under stimulated or neglected due to competition. However, it may also be possible that there is cross-epitope reactivity between rgp160 and FliC, and new regions of anti-gp160 reactivity are actually due to anti-FliC antibodies. Although this cannot be formally excluded here it may be unlikely due to the low nature of homology between antigen (gp160) and adjuvant (FliC). Nevertheless, these results suggest that detailed study of antibody responses in mice receiving pFliC(-gly) are warranted.

### 3.6. gp160 and p24gag DNA and Protein Vaccinations; Cellular Responses

**Figure 8:**
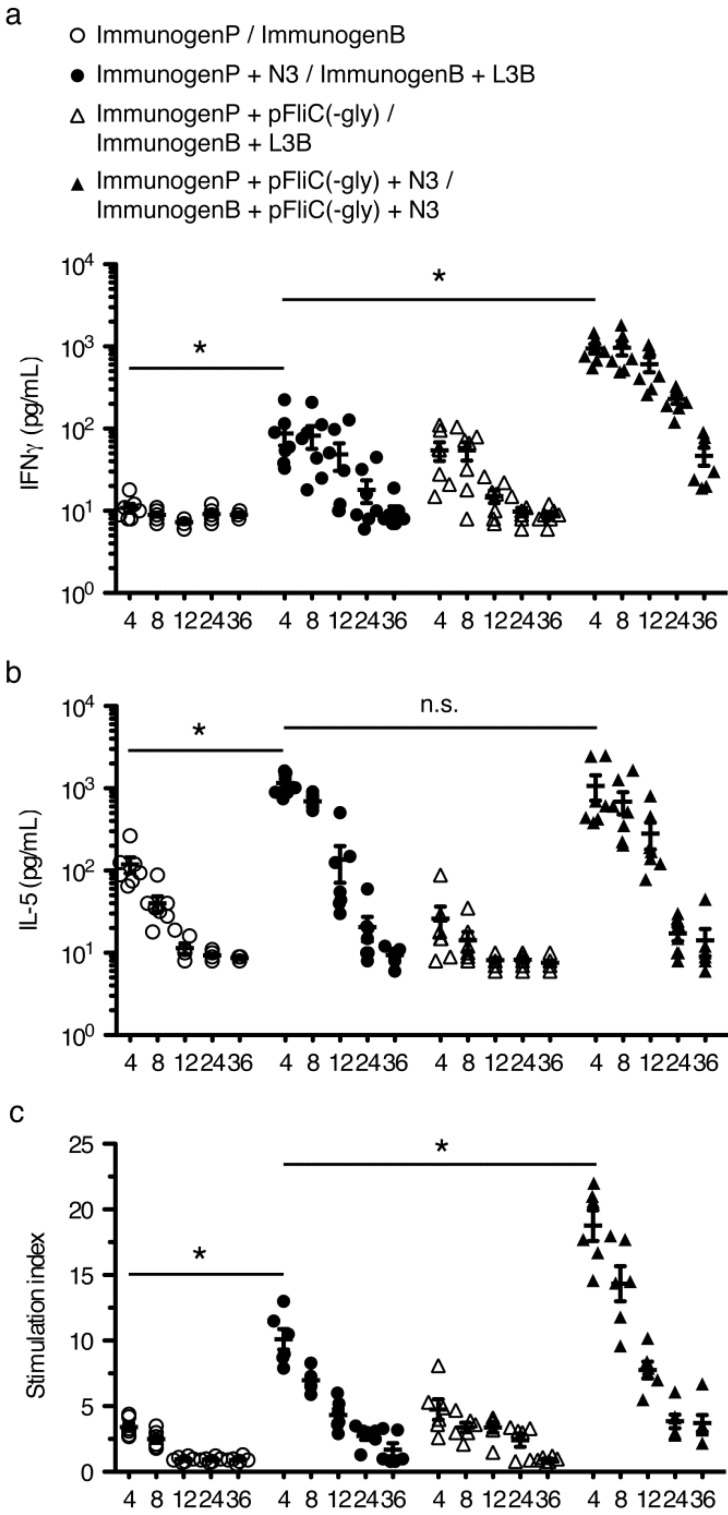
Kinetic analysis of T cell responses to immunizations with gp160 with and without adjuvant combinations. (**a**) Anti-mIFNγ ELISA was performed on cells restimulated with rgp160. Values shown were adjusted for baseline values seen using identical stimulations using splenocytes from naive mice. Priming (ImmunogenP, plasmids) and boosting (ImmunogenB, rec proteins) groups are shown in the key. Immunization details are listed in Table 2; (**b**) Anti-mIL-5 ELISA was performed on cells restimulated with rgp160. Values shown were adjusted for baseline values seen using identical stimulations using splenocytes from naïve mice; (**c**) Proliferative response to stimulation with rgp160 defined as stimulation index relative to identical stimulations using splenocytes from naïve mice. Statistical analyses were conducted using a two-tailed unpaired Student *t* test. * Differences of the responses between compared groups at week 4 after final boost defined as *p* ≤ 0.05 were considered significant. n.s. = non-significant. Comparisons between groups with the HIV-1 antigens were performed by using the non-parametric Mann-Whitney U test with Bonferroni correction, *p* < 0.05 was considered significant.

To study T cell immune responses to DNA-prime/protein-boost i.na. immunizations we chose to assay standard cytokines associated with Th1-like (IFNγ) or Th2-like (IL-5) populations. Responses to gp160 were assayed following individual splenocytes harvesting at 4, 8, 12, 24, and 36 weeks after final boost and restimulation with rgp160. Observed response trends were similar to those seen when studying antibody responses. Addition of N3 to pgp160 vaccinations followed by L3B protein boostings lead to clear and significant IFNγ, IL-5, and proliferative responses over mice immunized without adjuvant (Figure 8a–c). The IFNγ and proliferative responses could be further enhanced by the addition of pFliC(-gly) but not IL-5 (Figure 8a–c) demonstrating the ability of pFliC(-gly) to act as an adjuvant but with a propensity to strengthen Th1-like responses.

**Figure 9:**
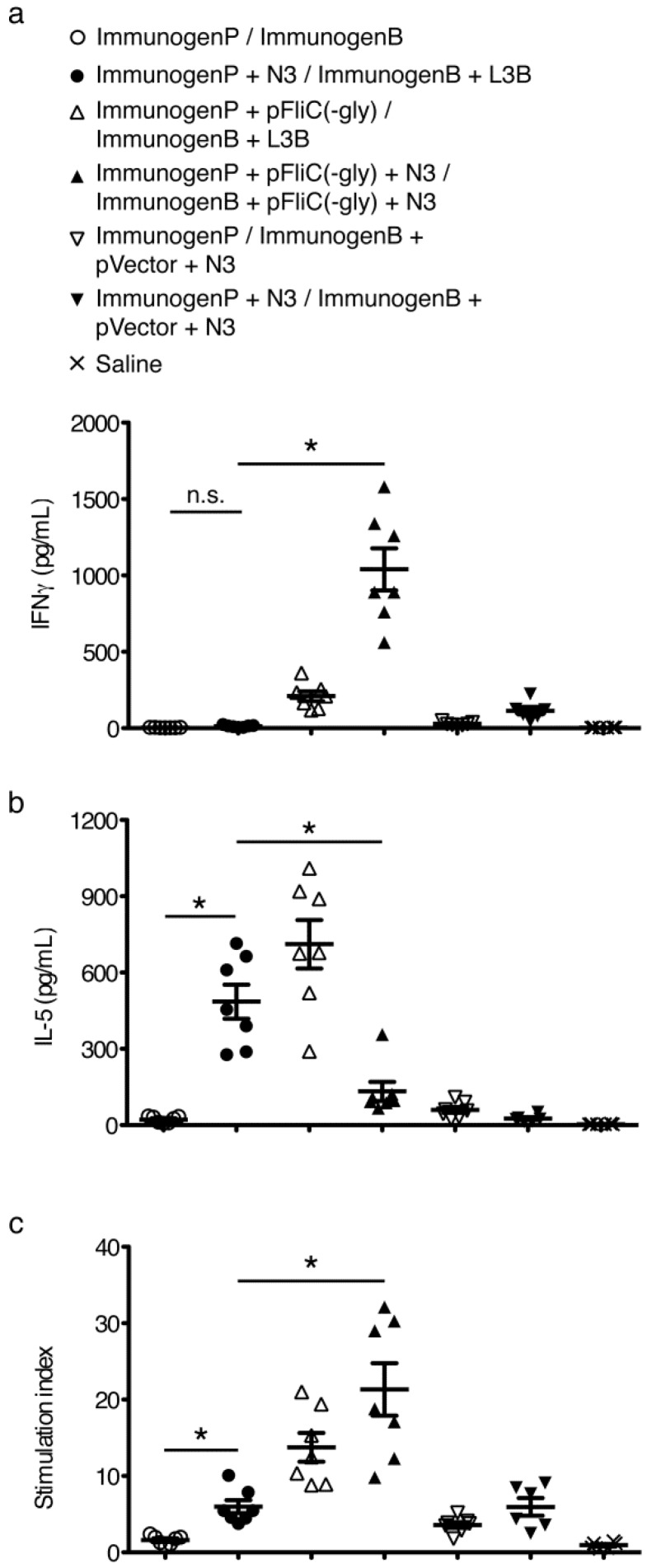
Analysis of T cell responses to immunizations with p24gag with and without adjuvant combinations. (**a**) Anti-mIFNγ ELISA was performed on cells restimulated with p24gag. Values shown were adjusted for baseline values seen using identical stimulations using splenocytes from naïve mice. Priming (ImmunogenP, plasmids) and boosting (ImmunogenB, rec proteins) groups are shown in the key. Immunization details are listed in Table 2; (**b**) Anti-mIL-5 ELISA was performed on cells restimulated with p24gag. Values shown were adjusted for baseline values seen using identical stimulations using splenocytes from naïve mice; (**c**) Proliferative response to stimulation with p24gag defined as stimulation index relative to identical stimulations using splenocytes from naïve mice. Statistical analyses were conducted using a two-tailed unpaired Student *t* test. * Differences of the responses between compared groups at week 4 after final boost defined as *p* ≤ 0.05 were considered significant. n.s. = non-significant. Comparisons between groups with the HIV-1 antigens were performed by using the non-parametric Mann-Whitney U test with Bonferroni correction, *p* < 0.05 was considered significant.

To study the T cell immune responses to p24gag individual splenocytes were harvested at 4 weeks after final boost and restimulated with rp24gag. Interestingly, the addition of N3 to p24gag vaccinations followed by L3B protein boostings only lead to clear and significant increases in IL-5 and proliferative responses compared to mice immunized without adjuvant (Figure 9a–c). Increases in IFNγ production were not seen. IFNγ and proliferative responses could be significantly enhanced by the addition of pFliC(-gly) but the IL-5 responses gained by use of N3 were suppressed by the addition of pFliC(-gly) (Figure 9a–c).

Interestingly the abilities of N3 and pFliC(-gly) to act as adjuvants did not completely overlap and, in one combination, even counteracted the other. In our immunizations the secreted antigen gp160 with N3 promoted a somewhat Th2-like response (including IFNγ) which was further enhanced by the addition of pFliC(-gly). Similar results were seen with the intracellular antigen p24gag where addition of pFliC(-gly) promoted IFNγ responses [27,28,29,30,31]. However, with p24gag the pFliC(-gly) addition actually suppressed IL-5 responses.

Together these results suggest that N3 has the ability to promote Th2-like adjuvant effects (IL-5 and proliferation) to extracellular and intracellular antigens whereas the effects of pFliC(-gly), which were greatly dependent on the presence N3, promoted Th1-like adjuvant effects (IFNγ, proliferation) sometimes at the expense of Th2-like responses (IL-5). Why these effects were dependent on the “location” of the antigen is unknown. However, it does suggest that with complex antigen/adjuvant mixtures that we are unable to predict exactly how they will affect adaptive immune responses.

### 3.7. Immune Activation Potential of Adjuvants

**Figure 10:**
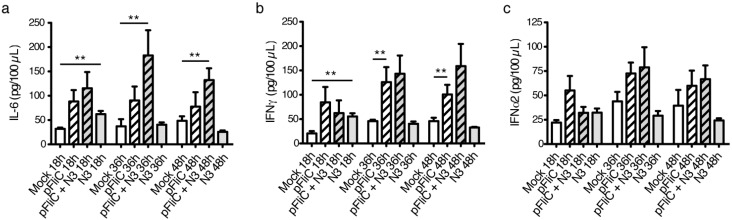
Cytokines produced after intranasal adminstration of adjuvant combinations. Kinetic analysis of (**a**) IL-6, (**b**) IFNγ, and IFNα2 (**c**) at 18, 36, and 48 h by ELISA using nasal wash samples. Mock adjuvant shown as White bars, use of pFliC(-gly) as Striped Bars, and N3 as Grey bars (*n* = 5 mice/group). Data is expressed as the calculated mean ± SEM. Statistical analyses were conducted using a two-tailed Mann-Whitney test. ** Differences relative compared groups defined as *p* ≤ 0.005 were considered significant.

To determine the types of inflammatory factors elicited intranasally by the adjuvants used we performed nasal mucosal washings at various time points after adjuvant delivery. We detected increases in the inflammatory cytokines IL-6, IFNα, and IFNγ at 18 to 48 h post-nasal adjuvant administration compared to nasal saline exposure (Figure 10a–c). Significant increases in IL-6 and IFNγ were elicited by N3 use at 18 hours post exposure while significant increases in IL-6 was elicited using a combination of pFliC(-gly) and N3 only at later time points (Figure 10a). These increases in IL-6 were dependent on the use of N3 with pFliC(-gly) but were not due to N3 or pFliC(-gly) alone.

### 3.8. Longevity of Immune Responses

Currently, it is still likely that several alternative prime-boost combinations will need to be tested to identify the most promising vaccine/adjuvant and vaccine design regimes for HIV-1 vaccines [32,33,34].

There are several important aspects concerning the flexibility associated with HIV-vaccine antigen development: first, the selection of immunogens and adjuvants. In this study, and in several others, DNA-plasmids should be selected that have long-lasting stability and allow persistence as stable antigen-expressing vectors. Second, production of DNA-plasmids, is today quite efficacious and can easily be performed at large-scale. Third, DNA-plasmids are attractive due to their great adaptability, and modifications in expression efficacy, gene exchange, or other desired modification is today easy to carry out.

Recombinant HIV-proteins as vaccine antigens are instead more of a challenge, especially when it comes to production and expression of such delicate proteins like the HIV-1 outer envelope. If they need to structurally mimic the envelope spikes found at the surface of HIV-1 primary isolates they need to be produced and maintained as multimeric, glycosylated envelope proteins. This production is not a trivial matter, and search for the ideal HIV-1 envelope vaccine candidate is still an unsolved issue [32,33]. Finally, the stable storage of sensitive recombinant proteins antigens is likely more of a problem. Thus a potent and safe adjuvant, formulated with obtainable amounts of quality antigen, may be a critical way to use these immunogenic proteins as vaccine candidates. In this study, we chose the recombinant baculovirus expressed HIV-1 gp160 due to its modest immunogenicity, its fair degree of glycosylation and trimeric structure and the content of both gp41 and gp120 envelope proteins [16,18].

From an immunological perspective, immunizing with a DNA plasmid with its endogenous *in vivo* expression of the HIV-antigen and especially of a highly glycosylated, conformation-sensitive antigens as HIV-1 gp120 is an attractive technology. Much of the trouble of production, safe storage and efficient administration of a neutralization antibody-inducing multimeric protein may then be reduced or avoided [35]. 

The durability of protective immune responses can often be enhanced by broadening antigen recognition ability through enhanced antigen delivery, enhanced antigen uptake, and/or prolonged antigen exposure [36,37]. Furthermore, by triggering several innate immune recognition pathways such as multiple Toll-like receptors (with DNA-plasmid CpG motifs, (TLR9), with FliC expression (TLR5), cytoplasmic DNA sensors [38,39] and increased induction of cell death using surfactant adjuvants in conjunction with antigen presentation as with cationic lipids more potent immune enhancement may be obtained [40]. In this study, we endeavored to optimize the immunological outcome (strength and longevity) of these vaccine technologies by methodologically studying optimal route delivery for adjuvants with DNA-plasmid vaccination followed by the application of this knowledge to a heterologus prime-boost regime using HIV-1 antigens. Importantly, we show that in the HIV-1 study group (group No.4) receiving potentially the broadest HIV-1 antigen variants and the most complex adjuvant combination we obtained the most long-lasting humoral and cell mediated immune responses.

### 3.9. Impact of Adjuvant on Local Innate Immunity

Certain studies have described the induction of innate and adaptive immune reactivity when immunogens and adjuvant have been given to mice [41,42,43]. In the current studies we have performed analyses on how the cationic lipid-based N3 adjuvant, the DNA-plasmid-based FliC (-gly) adjuvant, and the combination of both together influence the local and systemic innate and adaptive immune responses. The data indicate that the adjuvants stimulate the production of pro-inflammatory IL-6 and interferon (IFN), two cytokines described promote innate immunity and B cell activation [44,45].

Each of the adjuvants are likely to contribute, in their own way, to immune activation, and lipid-based adjuvants (such as the N3) have been observed to induce cell death (as seen with N3 *in vitro*, data not shown) and local inflammation. This would provide danger signals to attract antigen-presenting cells, stimulate antigen uptake, and induce dendritic cell maturation. For instance, cell death and endogenous DNA release would be able to function as an endogenous adjuvant capable of supporting IL-6 release and a T-helper type 2 response [41,45,46]. Inflammatory cytokine patterns, accompanied higher antibody titers have been reported by Valesi *et al.*, when using the MF59 lipid emulsion adjuvant and influenza vaccine given to mice [47]. Similar results in inflammatory cytokine production with the N3 lipid adjuvant and HIV antigens were also observed in our work.

DNA-plasmid vaccination used to express antigen and adjuvant proteins can trigger TLR systems and promote inflammatory cytokine production (such as IL-6) [48]. Intranasal recombinant FliC polypeptide has also been observed to induce inflammatory cytokines including IL-6 [49]. Interestingly, the induction of IL-6 secretion in mucosal stimulation has been reported to improve transepithelial transport over mucosal epithelial barriers [50]. This effect of IL-6 may explain the enhanced mucosal immune responses we observe using mucosally delivered pFliC(-gly). However, the ability of FliC to promote mucosal adaptive immunity is complex and appears to also involve the production of numerous other factors not studied here such as IL-17, IL-22, and IL-23 [51,52].

Our observations of mucosal, antigen-specific IgA elicited by intra-nasal immunization indicates presence of a Th2-type response with N3 use while IFNγ production detected in the nasal washes of pFliC(-gly) immunized animals suggest a Th1-type response. Our results also suggest that these responses need not be mutually exclusive. Adjuvant emulsions such as MF59 and similar products applied mucosally have been shown to act as Th2-type adjuvants, and addition of additional stimuli such as CpGs, and other TLR agonists have been observed to skew immune responses towards a Th1-type or a balanced Th1/Th2-type immune pattern [38,53]. Here, we show data that immune responses induced using a Th2-enhancing lipid adjuvant (N3) may be modified by the addition of a TLR-agonist and inflammasome trigger (pDNA, FliC), to also promote a Th1-type (IFNγ) response when administered nasally.

## 4. Conclusions

Adjuvant choice during DNA vaccine development will depend on formulation relative to method of delivery, the recipient, the protective antigens used, as well as the desired induction of immunity at the portal of infection. As the detection of flagellin by innate immune receptors is evolutionarily conserved, it has the potential to be easily used in many species without the need to isolate and prepare species-specific adjuvants such as cytokines [27,28,29,30,31]. These unique properties as well as its ability to promote both humoral and cellular responses to co-delivered antigens by multiple routes without a need for manipulating the antigen indicate that it works as an easy and efficient adjuvant to improve non-living non-replicating DNA vaccines.

Finally, in this work, all immunizations with HIV-antigens were delivered as heterologous prime boost immunizations. Antigens are presented both through endogenous expression and as recombinant soluble proteins to the immune system to provide the antigenic regions or epitopes with greater variation than in a homologous immunization. With this approach we demonstrate that cationic lipids formulated with plasmid FliC-DNA and plasmid HIV-DNA, followed by cationic lipids formulated with plasmid FliC-DNA and recombinant HIV-1 proteins (study group 4) elicited the longest-lived immunity and broadest antigen epitope recognition.

## Abbreviations

TLR: Toll-like Receptor
NLRC4: Nod-like Receptor family CARD domain-containing protein 4
Naip5: Neuronal apoptosis inhibitory protein 5
HIV-1: Human Immunodeficiency Virus-1
